# Acute sleep deprivation alters hypothalamic neuroinflammatory signaling independent of traumatic brain injury in the mouse

**DOI:** 10.1093/sleepadvances/zpag076

**Published:** 2026-07-13

**Authors:** Francois Labadie, Grant S Mannino, Jonathan Lifshitz, Rachel K Rowe

**Affiliations:** Department of Integrative Physiology, University of Colorado Boulder, Boulder, CO, United States; Department of Integrative Physiology, University of Colorado Boulder, Boulder, CO, United States; Stanford University School of Medicine, Neurosciences Interdepartmental Program, Stanford, CA, United States; Physical Medicine and Rehabilitation, University of Michigan, Ann Arbor, MI, United States; University of Michigan Concussion Center, Ann Arbor, MI, United States; Department of Integrative Physiology, University of Colorado Boulder, Boulder, CO, United States

**Keywords:** concussion, sleep disruption, sleep fragmentation, neuroinflammation, gene expression, microglia, cytokines

## Abstract

**Study Objectives:**

Traumatic brain injury (TBI) is frequently accompanied by sleep disturbances and neuroinflammatory responses that contribute to long-term neurological dysfunction. Sleep deprivation commonly occurs during the acute post-injury period, yet its influence on early neuroinflammatory signaling remains poorly understood.

**Methods:**

Here, we investigated how acute sleep deprivation alters inflammatory gene expression following experimental TBI in mice. Adult male C57BL/6J mice underwent midline fluid percussion injury (mFPI) or sham surgery and were subsequently exposed to 6 hours of gentle-handling sleep deprivation or undisturbed sleep. Following the manipulation period, the hypothalamus, thalamus, hippocampus, motor cortex, and primary somatosensory cortex (S1BF) were collected for transcriptional profiling using the NanoString nCounter Mouse Neuroinflammation Panel.

**Results:**

Sleep deprivation emerged as the dominant driver of transcriptional changes across brain regions at 6 hours post-injury, whereas TBI alone produced comparatively modest effects. Transcriptional responses to sleep deprivation were highly localized to the hypothalamus, where 23 genes were differentially expressed, with minimal responses observed in other regions. Differentially expressed genes included Mapk1, Jun, Map2k1, and Il1rap. Gene set enrichment analysis demonstrated coordinated suppression of MAPK/ERK signaling, NF-κB-mediated inflammatory pathways, cytokine and chemokine signaling, complement activation, and pathways related to cytoskeletal remodeling and neuronal signaling. The combination of TBI and sleep deprivation did not produce a synergistic transcriptional response, suggesting that sleep deprivation dominates early neuroinflammatory signaling.

**Conclusions:**

These findings identify acute sleep deprivation as a potent modulator of early neuroimmune signaling and highlight sleep as a potential therapeutic target following TBI.

**Statement of Significance**

Sleep disturbances are common immediately after traumatic brain injury, yet the biological consequences of acute sleep loss during this vulnerable period remain poorly understood. This study identifies acute sleep deprivation as a major driver of early neuroimmune signaling, producing stronger transcriptional effects than brain injury itself at the examined time point. Importantly, these responses were highly localized to the hypothalamus, a brain region critical for sleep–wake regulation and neuroimmune integration. These findings challenge the assumption that early post-injury inflammation is driven primarily by the injury alone and instead support sleep loss as an active contributor to the post-traumatic molecular environment. Protecting sleep after brain injury may therefore represent a clinically relevant therapeutic strategy.

## Graphical Abstract

**Graphical Abstract:**
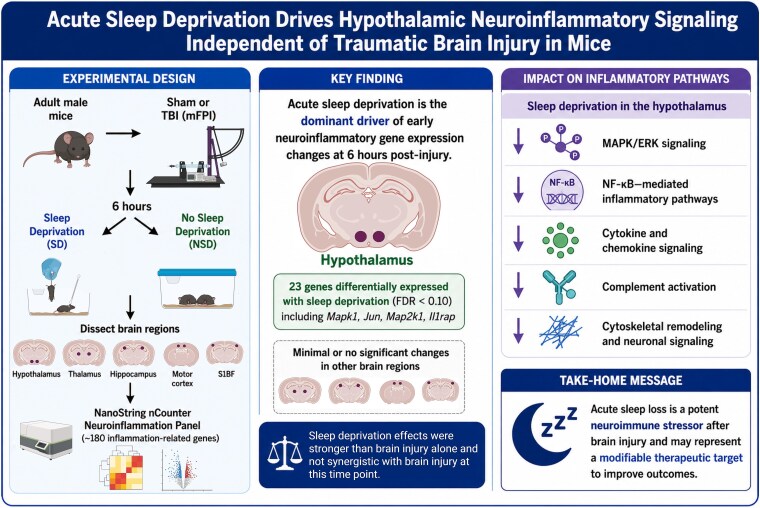

## Introduction

Traumatic brain injury (TBI) is a major public health concern affecting an estimated 70 million individuals globally and is associated with long-term cognitive, behavioral, and sleep disturbances [1–5]. Beyond the primary mechanical insult, TBI initiates a secondary injury cascade characterized by neuroinflammation, glial activation, and cytokine signaling that contributes to ongoing tissue damage and neurological dysfunction [6–8]. This secondary phase evolves over hours to days and represents a critical window during which modifiable physiological factors may influence recovery trajectories [8].

Neuroinflammation following TBI is driven by coordinated responses of microglia and astrocytes, which regulate cytokine production, immune signaling, and cellular stress pathways [9–11]. These processes are highly dynamic and involve complex interactions across multiple cell types and signaling networks, which can be captured through transcriptional profiling approaches [12, 13]. Importantly, the neuroinflammatory response to TBI is not uniform across the brain but instead exhibits marked regional heterogeneity, reflecting differences in cellular composition, connectivity, and functional specialization [13]. Understanding how distinct brain regions respond to injury at the molecular level is therefore critical for identifying mechanisms that drive both vulnerability and recovery.

Sleep disturbances are among the most commonly reported consequences of TBI in patient populations, including during the acute post-injury period; however, the underlying neurobiological mechanisms remain poorly understood [4, 5, 14, 15]. Sleep and the immune system are tightly interconnected, with bidirectional interactions mediated, in part, by cytokine signaling pathways [16–19]. Within hours, sleep deprivation induces neuroinflammatory signaling, including increased expression of cytokines, such as interleukin-1β and tumor necrosis factor-α, as well as activation of intracellular pathways, such as NF-κB and MAPK signaling [20, 21]. Microglia play a central role in these processes, contributing to both inflammatory signaling and the regulation of sleep homeostasis [22, 23]. Together, these findings establish sleep loss as a potent modulator of neuroimmune function.

In prior work using the same experimental paradigm, we demonstrated that 6 hours of acute sleep deprivation immediately following diffuse TBI does not impair early neurological recovery, despite observed disruption of sleep–wake behavior during the acute post-injury period [4, 5, 24]. However, whether this same acute sleep loss influences underlying neuroinflammatory signaling has not been examined. Despite strong evidence that both TBI and sleep deprivation independently influence neuroinflammatory signaling, their interaction during the acute post-injury period remains poorly understood. Importantly, these processes operate on distinct temporal scales. In experimental models of diffuse TBI, neuroinflammatory signaling evolves over hours-to-days post-injury, whereas sleep deprivation can elevate inflammatory cytokine concentrations within the first few hours (≤6 hours), highlighting distinct but overlapping temporal dynamics [8, 25–29]. This temporal mismatch suggests that sleep loss may act as an early modulator of neuroimmune signaling following injury, potentially shaping subsequent neuroinflammatory pathways as TBI-induced responses unfold. However, whether acute sleep deprivation alters early neuroinflammatory transcriptional responses following TBI has not been systematically examined.

In addition, it remains unclear whether the effects of sleep deprivation on neuroinflammatory signaling are uniformly distributed across the brain or preferentially localized to specific regions. The hypothalamus is of particular interest in this context, as it serves as a central hub for sleep–wake regulation, circadian timing, and neuroimmune integration [30, 31]. Neuronal populations within the hypothalamus, including hypocretin-producing neurons and sleep-promoting circuits, are highly sensitive to both inflammatory signaling and sleep–wake state, positioning this region as a potential interface linking behavioral state to neuroimmune responses [32–34]. Cytokine signaling has also been shown to disrupt circadian regulation through effects on hypothalamic clock gene expression, further highlighting the susceptibility of this region to inflammatory perturbations [35, 36].

We therefore tested the hypothesis that acute sleep deprivation significantly alters early neuroimmune transcriptional responses following TBI and that these effects are region-specific. Mice were subjected to midline fluid percussion injury (mFPI) or sham injury and subsequently exposed to acute sleep deprivation or undisturbed conditions. We quantified inflammatory gene expression across multiple brain regions, including the hypothalamus, thalamus, hippocampus, and cortical areas. This approach enables a systems-level analysis of how sleep loss and injury interact to shape early neuroimmune signaling across the mouse brain. Ultimately, this work provides mechanistic insight into how acute sleep loss alters early neuroimmune transcriptional responses after TBI and identifies candidate molecular pathways that may contribute to injury and recovery processes.

## Materials and methods

### Rigor

All animal studies were conducted in accordance with the guidelines established by the Institutional Animal Care and Use Committee (IACUC) at the University of Kentucky and the National Institutes of Health (NIH) guidelines for the care and use of laboratory animals. Studies are reported following the Animal Research: Reporting In Vivo Experiments (ARRIVE) guidelines [37]. Analysis of inflammatory data was performed by investigators blinded to experimental conditions.

### Animals

Adult (12-16 weeks old) male C57BL/6J mice were used. All mice were obtained from Jackson Laboratories (Bar Harbor, ME). Mice were group-housed for the duration of the study and maintained on a 12-hour light:dark cycle at an ambient temperature of 25°C ± 1°C. All mice were acclimated for a minimum of 7 days following shipping prior to initiation of data collection. Mice were fed a normal diet of standard rodent chow, and food and water were available *ad libitum*.

**Figure 1:**
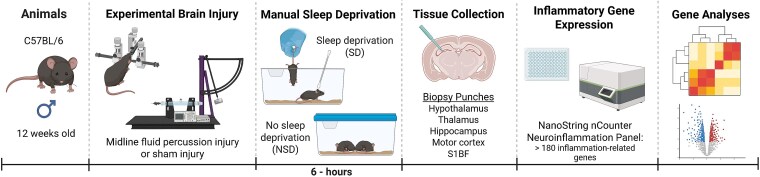
Experimental design. Adult male mice were subjected to midline fluid percussion injury (mFPI) or sham injury. Immediately following TBI, mice were assigned to either manual sleep deprivation (SD) or undisturbed sleep (no sleep deprivation; NSD) conditions. Sleep deprivation was performed using gentle handling to prevent sleep. Six hours after initiation of the sleep manipulation, brain tissue was collected. Discrete brain regions including the hypothalamus, thalamus, hippocampus, motor cortex and primary somatosensory cortex (S1BF) were dissected using tissue biopsy punches for gene expression analysis. Inflammatory gene expression was quantified using the NanoString nCounter neuroinflammation panel (185 inflammation-related genes). Downstream analyses included differential expression testing, heatmap visualization, and pathway enrichment analyses to assess the effects of traumatic brain injury, sleep deprivation, and interactions across brain regions.

Prior to study initiation, mice were randomly assigned to an experimental group (sham sleep deprivation *n* = 3, sham no sleep deprivation *n* = 3, TBI sleep deprivation *n* = 3, TBI no sleep deprivation *n* = 3). Each mouse contributed region-specific tissue samples with a total of ~60 region-specific samples used for downstream analyses. All surgeries were performed under anesthesia with isoflurane, and exclusion criteria were predetermined: mice that had visible signs of distress were euthanized and excluded from the study (*n* = 0). Predetermined inclusion criteria included a righting reflex time of >300 seconds. See Figure 1 for the complete study design.

All experimental procedures were performed during the light period. Craniectomy and injury hub implantation surgeries were completed 24 hours prior to injury. On the experimental day, sham and mFPI procedures were initiated at zeitgeber time (ZT) 0 and finished by ZT2. Animals were processed sequentially, and sleep deprivation or no sleep deprivation conditions began immediately following each injury or sham procedure. Animals were euthanized immediately following completion of the 6-hour experimental period (initiated at ZT6). Importantly, each animal was collected at a consistent interval following injury or sham treatment. The timing of injury, sleep manipulation, and tissue collection was held consistent across cohorts and balanced across experimental groups to minimize potential circadian influences on gene expression.

### Midline fluid percussion injury

Mice were anesthetized with 5% isoflurane delivered in 100% oxygen for approximately 5 minutes, after which they were secured in a stereotaxic frame and maintained under a continuous flow of 2.5% isoflurane in 100% oxygen delivered via a nose cone. Ophthalmic ointment was applied to prevent corneal drying, and the surgical field was disinfected using alternating applications of betadine and ethanol. A midline scalp incision was performed to expose the skull surface. A 3-mm diameter craniectomy was trephined along the sagittal suture, midway between bregma and lambda. An injury hub constructed from a modified Luer-Loc needle hub was secured over the craniectomy using cyanoacrylate adhesive followed by dental acrylic (Hygenic Corp., Akron, OH). The hub was filled with sterile saline and sealed with a custom cap fashioned from a modified syringe tip to prevent air exposure and contamination.

Mice were re-anesthetized 24 hours later using 5% isoflurane in 100% oxygen for approximately 3 minutes. The protective cap was removed, and the dura was visually inspected to confirm integrity. The hub was then refilled with saline and connected via extension tubing to the fluid percussion injury (FPI) device (Custom Design and Fabrication, Virginia Commonwealth University, Richmond, VA). Upon return of a toe-pinch reflex, the pendulum was released, delivering a fluid pulse to the intact dura, using parameters consistent with our previously published protocols [5, 38–40]. Sham control animals underwent identical surgical preparation and device attachment, but did not receive a fluid pulse. Following injury or sham procedure, animals were briefly re-anesthetized, the surgical site was cleaned, and the incision closed with sutures. Topical antibiotic ointment (bacitracin) was applied, and mice were returned to a thermally regulated recovery cage.

### Sleep deprivation

Immediately following TBI, mice were randomly assigned to a sleep deprivation or no sleep deprivation (control) group. Control mice were transferred to the vivarium housing room, group housed in standard shoebox cages (4–5 mice per cage), and remained undisturbed for the duration of the experimental period. Mice in the sleep deprivation group were transferred to a separate experimental room, group housed in standard open-top shoebox cages (4–5 mice per cage), and received continuous sleep deprivation for 6 hours beginning immediately after the TBI or sham procedure. Sleep deprivation was accomplished using a minimally stressful gentle-handling method, as previously described [24]. For sleep deprivation, mice were under constant observation and the cage was tapped or the mouse was gently touched with a cotton applicator stick when visible signs of sleep were present [41].

### Tissue preparation

After 6 hours of sleep deprivation, brain tissue was collected for region-specific gene expression analysis. Mice were euthanized by intraperitoneal injection of Euthasol and transcardially perfused with ice-cold 1× phosphate-buffered saline (PBS) to remove circulating blood and minimize peripheral RNA contamination. Brains were rapidly extracted, placed into a pre-chilled brain matrix, and sectioned into 50-μm coronal slices using sterile razor blades.

Discrete brain regions of interest (hypothalamus, thalamus, hippocampus, motor cortex, and primary somatosensory cortex [S1BF]) were isolated using a sterile tissue biopsy punch. For each animal, all brain regions were collected separately and processed as independent biological samples. No tissue pooling was performed across animals at any stage of sample collection, processing, or NanoString analysis. For the thalamus, two punches were collected from the same animal (e.g., anterior and posterior thalamic regions) and combined to generate a single regional sample for downstream analysis. Tissue punches were collected into sterile, RNase-free microcentrifuge tubes, snap-frozen in methylbutane cooled on dry ice to preserve RNA integrity, and stored at −80°C until RNA extraction and NanoString nCounter analysis.

### Gene expression analysis

Total RNA was extracted from region-specific brain tissue punches by homogenizing frozen samples in QIAzol Lysis Reagent (QIAGEN, Hilden, Germany) followed by purification using the RNeasy Mini Kit (QIAGEN), according to the manufacturer’s instructions. RNA concentration and integrity were assessed using the Agilent 2100 Bioanalyzer (Agilent Technologies, Santa Clara, CA).

Gene expression profiling was performed using the NanoString nCounter Analysis System with a custom mouse neuroinflammation-focused CodeSet (NanoString Technologies) targeting 185 endogenous genes involved in neuroimmune signaling. The CodeSet included genes associated with pro-inflammatory cytokine signaling, chemokine signaling, innate immune activation, complement pathways, microglial and astrocytic responses, intracellular stress signaling, and anti-inflammatory and immune-regulatory pathways. The CodeSet also included NanoString-provided synthetic positive control probes, negative control probes, binding controls, and purification controls for technical quality assessment and normalization. This design enabled assessment of both inflammatory activation and transcriptional programs involved in immune regulation and resolution. For each sample, 100 ng of total RNA was hybridized with reporter and capture probes according to NanoString’s standard hybridization protocol.

Sample quality was assessed using NanoString platform-specific quality control metrics extracted from raw RCC files. All samples demonstrated high imaging efficiency (100% fields of view counted), robust positive control performance with expected linearity across spike-in concentrations, and low background signal based on negative controls. Binding densities were near the recommended range, and all samples met overall quality control criteria for inclusion in downstream analyses. A small number of region-specific samples did not meet quality control criteria and were excluded from downstream analyses, resulting in fewer than three biological replicates for select brain regions.

Raw counts were normalized using NanoString nSolver software. Data were first normalized to NanoString-provided synthetic positive control probes consisting of ERCC RNA spike-in controls present at known concentrations to account for technical variability. Data were subsequently normalized to the geometric mean of housekeeping genes to control for differences in RNA input and sample quality. Background signal was estimated using NanoString negative control probes and subtracted from normalized counts. Following sample-level quality control and normalization, all genes contained within the CodeSet were retained for downstream analyses. No additional expression-based filtering thresholds or minimum detection-frequency criteria were applied prior to differential expression testing. Therefore, all 185 targeted genes were evaluated across brain regions using the same analytical pipeline.

### Differential gene expression

Differential expression analyses were performed to assess the effects of sleep deprivation (SD), TBI, and their interaction across brain regions. Normalized NanoString counts were analyzed in R (version 4.5.0; R Foundation for Statistical Computing) using linear modeling approaches implemented for a 2 × 2 factorial design comparing four experimental groups: Sham-no SD, Sham-SD, TBI-no SD, and TBI-SD.

Linear models were used to estimate log2 fold changes and statistical significance for the main effects of SD, TBI, and their interaction. *P*-values were corrected for multiple comparisons using the Benjamini-Hochberg false discovery rate (FDR) method. Genes with FDR-adjusted *p* < .10 were considered significantly differentially expressed, without the application of a minimum log2 fold change threshold. Log2 fold changes are reported to indicate the magnitude and direction of expression changes.

Hierarchical clustering heatmaps were generated from normalized gene expression values using row-wise Z-score transformation. Genes were clustered using Euclidean distance and complete linkage to visualize coordinated expression patterns across experimental groups. Volcano plots were generated using log2 fold change and -log10(FDR) values to visualize the magnitude and significance of differential gene expression. All figures were generated in R using the ggplot2 and pheatmap packages.

### Pathway enrichment and network analysis

Pathway enrichment analyses were performed using gene set enrichment analysis (GSEA) to assess coordinated biological responses across the NanoString panel. All genes were ranked based on moderated differential expression statistics, and enrichment was assessed using the fgsea R package with Kyoto Encyclopedia of Genes and Genomes (KEGG) pathway annotations. Pathway significance was determined by normalized enrichment scores (NES) and FDR-adjusted *p*-values.

Significantly enriched pathways were grouped into broader functional categories related to immune signaling, cytokine activity, complement activation, and MAPK-mediated inflammatory signaling. Leading-edge genes contributing to pathway enrichment were identified and used to construct mechanistic interaction networks highlighting key regulatory nodes involved in sleep deprivation- and TBI-associated inflammatory signaling.

## Results

### Sleep deprivation induces region-specific inflammatory transcriptional responses across brain regions

**Figure 2:**
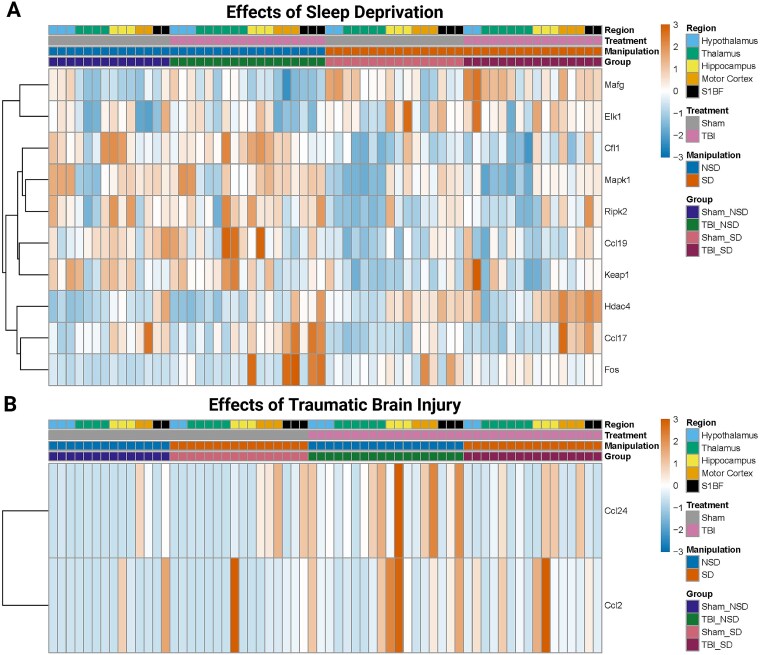
Global transcriptional effects of sleep deprivation and traumatic brain injury (TBI) across brain regions. (A) Sleep deprivation (SD) effect. Heatmap showing genes differentially expressed with sleep deprivation (FDR < 0.10) across all brain regions. Rows represent genes and columns represent individual samples. Expression values are row-wise z-score–normalized across samples, where 0 represents the mean expression level for each gene, positive values indicate higher relative expression, and negative values indicate lower relative expression. Samples are ordered by manipulation (no sleep deprivation [NSD], sleep deprivation [SD]), followed by treatment (Sham, TBI) and brain region (hypothalamus, thalamus, hippocampus, motor cortex, and primary somatosensory cortex [S1BF]). Annotation bars indicate experimental group, manipulation, treatment, and region. Sleep deprivation produced coordinated transcriptional patterns across samples, with region-specific effects that were most pronounced in the hypothalamus and minimal changes observed in other regions. (B) TBI effect. Heatmap showing genes differentially expressed with TBI (FDR < 0.10) across all brain regions. Expression values are row-wise z-score–normalized across samples. Samples are ordered by treatment (Sham, TBI), followed by manipulation (NSD, SD) and brain region. In contrast to sleep deprivation, TBI alone induced a comparatively limited transcriptional response at 6 hours post-injury. Color scale: Values represent row-wise z-score–normalized gene expression, with cooler colors indicating lower relative expression and warmer colors indicating higher relative expression across samples.

Hierarchical clustering of normalized gene expression values revealed distinct transcriptional patterns associated with sleep deprivation across brain regions (Figure 2A). Differential expression analysis using a factorial model (TBI × SD) identified sleep deprivation as the dominant driver of inflammatory gene expression, whereas the transcriptional effects of TBI alone were comparatively modest. Across all brain regions combined, sleep deprivation significantly altered the expression of multiple inflammatory genes (FDR < 0.10; Table 1), whereas few genes met the significance threshold for the main effect of TBI. Likewise, no genes met significance for the TBI × sleep deprivation interaction at the predefined false discovery rate threshold (FDR < 0.10).

**Table 1:** Differentially expressed genes associated with traumatic brain injury and sleep deprivation across brain regions

Brain region	Comparison	Gene	log2FC	*p* value	FDR
All regions	TBI vs Sham	Ccl24	1.187	.000164	0.0304
All regions	TBI vs Sham	Ccl2	1.872	.00073	0.0675
All regions	SD vs NSD	Ccl17	-0.95	4.24e-05	0.00486
All regions	SD vs NSD	Mapk1	-0.286	5.25e-05	0.00486
All regions	SD vs NSD	Hdac4	0.279	.000184	0.00871
All regions	SD vs NSD	Mafg	0.286	.000188	0.00871
All regions	SD vs NSD	Cfl1	-0.567	.000828	0.0298
All regions	SD vs NSD	Fos	1.276	.000968	0.0298
All regions	SD vs NSD	Ccl19	-0.911	.00118	0.0313
All regions	SD vs NSD	Ripk2	-0.276	.00323	0.0714
All regions	SD vs NSD	Keap1	-0.179	.00347	0.0714
All regions	SD vs NSD	Elk1	0.283	.0052	0.0963
Hypothalamus	SD vs NSD	Rps6ka5	-1.005	7.9e-05	0.00158
Hypothalamus	SD vs NSD	Mapk1	-0.924	8.3e-05	0.00158
Hypothalamus	SD vs NSD	Jun	0.967	8.5e-05	0.00158
Hypothalamus	SD vs NSD	Map2k1	-0.924	8.3e-05	0.00158
Hypothalamus	SD vs NSD	Cd4	-5.987	.00229	0.0221
Hypothalamus	SD vs NSD	Gnb1	0.177	.00229	0.0221
Hypothalamus	SD vs NSD	Plcb1	-0.914	.00229	0.0221
Hypothalamus	SD vs NSD	Map2k4	0.416	.00229	0.0221
Hypothalamus	SD vs NSD	Nr3c1	0.565	.00229	0.0221
Hypothalamus	SD vs NSD	Mef2d	-0.806	.00229	0.0221
Hypothalamus	SD vs NSD	Gnas	0.802	.00229	0.0221
Hypothalamus	SD vs NSD	Hdac4	0.487	.00274	0.0242
Hypothalamus	SD vs NSD	Cdc42	0.17	.00318	0.0263
Hypothalamus	SD vs NSD	C1qb	-0.526	.00364	0.0293
Hypothalamus	SD vs NSD	Prkcb1	-1.793	.00383	0.03
Hypothalamus	SD vs NSD	Pdgfa	0.52	.00548	0.0399
Hypothalamus	SD vs NSD	Il1rap	0.67	.00618	0.0424
Hypothalamus	SD vs NSD	Tollip	0.311	.00722	0.0488
Hypothalamus	SD vs NSD	Grb2	-0.271	.0108	0.0614
Hypothalamus	SD vs NSD	Cd55	0.809	.0119	0.0646
Hypothalamus	SD vs NSD	Mef2c	-1.684	.012	0.065
Hypothalamus	SD vs NSD	Cltc	0.141	.0187	0.0958
Hypothalamus	SD vs NSD	Ptk2	-0.351	.0196	0.0989
Thalamus	SD vs NSD	Fos	1.388	.000477	0.0883

Differential gene expression analysis was performed using linear modeling with empirical Bayes moderation. Genes with a false discovery rate (FDR) < 0.10 are reported. Analyses were conducted for traumatic brain injury (TBI vs Sham) and sleep deprivation (SD vs NSD) across all brain regions combined and within individual brain regions. log2FC indicates the log₂ fold change in gene expression between comparison groups.

When analyzed at the regional level, the magnitude of sleep deprivation–induced transcriptional changes differed across brain regions. The hypothalamus exhibited the largest number of differentially expressed genes, with 23 of approximately 185 genes significantly altered (12.8%), whereas only one gene (0.6%) was significantly altered in the thalamus and no genes met the significance threshold in the hippocampus, motor cortex, or S1BF under the same conditions.

Heatmap visualization also showed that sleep deprivation produced coordinated shifts in inflammatory gene expression across experimental groups, with clustering patterns reflecting both sleep deprivation status and brain region identity (Figure 2A and B). These data demonstrate that sleep deprivation induces region-specific inflammatory transcriptional responses, with the hypothalamus showing the greatest number of differentially expressed genes. Complete differential expression results for sleep deprivation, TBI, and their interaction across all regions are provided in Table 1.

To further isolate the effects of injury independent of sleep deprivation, we performed a secondary analysis restricted to mice with no sleep deprivation. This analysis identified a single differentially expressed gene, *Ccl24* (FDR = 0.087), indicating that TBI alone produced minimal transcriptional changes at this acute time point.

### Sleep deprivation selectively alters inflammatory gene expression in the hypothalamus

Because the global regional analysis indicated that sleep deprivation was the primary driver of transcriptional changes across the NanoString inflammatory gene panel, we next examined hypothalamic transcriptional responses in greater detail. A similar analysis in other brain regions was not possible due to the lack of differentially expressed genes. Differential expression analysis revealed a robust inflammatory gene expression response to sleep deprivation in the hypothalamus.

**Figure 3:**
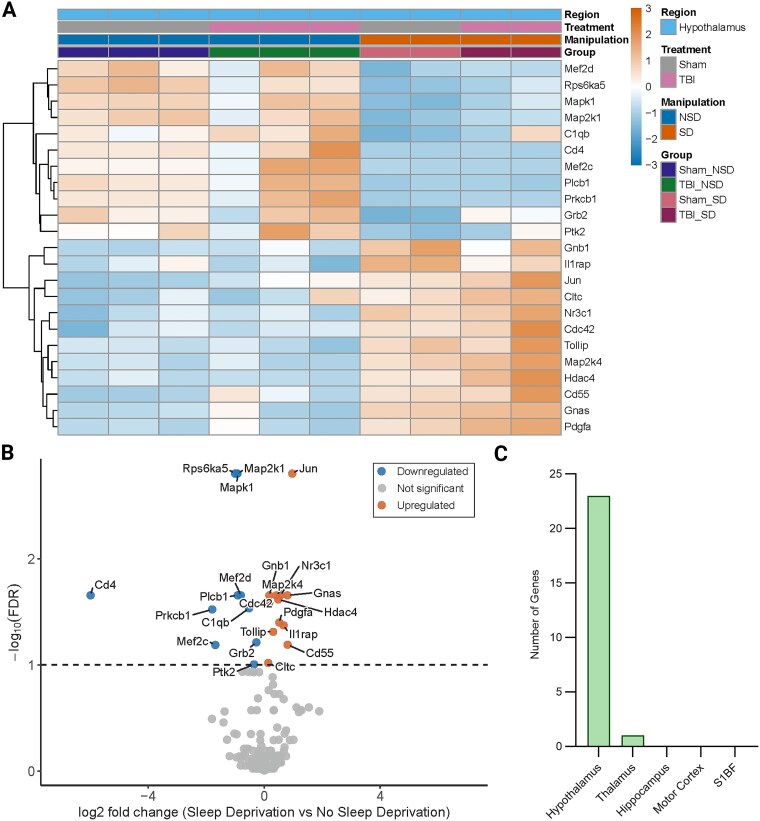
Sleep deprivation selectively alters inflammatory gene expression in the hypothalamus. (A) Hypothalamic differential gene expression. Heatmap of genes differentially expressed with sleep deprivation (FDR < 0.10). Rows represent genes and columns represent individual samples. Expression values are row-wise z-score–normalized across samples, where 0 indicates the mean expression level for each gene and positive and negative values indicate higher and lower relative expression, respectively. Samples are ordered by manipulation (no sleep deprivation [NSD], sleep deprivation [SD]), followed by treatment (Sham, TBI). Annotation bars indicate experimental group, manipulation, treatment, and region. Genes exhibit coordinated patterns of upregulation and downregulation, reflecting bidirectional regulation of inflammatory and signaling pathways. (B) Volcano plot of hypothalamic differential expression. Volcano plot showing log₂ fold change versus −log₁₀(FDR) for genes comparing SD to NSD conditions. Each point represents a gene. Genes meeting the significance threshold (FDR < 0.10) are colored by direction of differential expression, with blue indicating downregulated genes and warm colors indicating upregulated genes, while non-significant genes are shown in gray. The dashed line indicates the significance threshold (FDR = 0.10). Labeled genes represent those meeting the significance threshold. (C) Regional distribution of differential gene expression. Number of genes differentially expressed with sleep deprivation (FDR < 0.10) across brain regions. Differential expression was predominantly restricted to the hypothalamus (23 genes), with one gene identified in the thalamus and no significant changes detected in the hippocampus, motor cortex, or primary somatosensory cortex (S1BF).

Hierarchical clustering of genes significantly altered by sleep deprivation demonstrated coordinated transcriptional shifts between experimental groups (Figure 3A). Row-scaled heatmap visualization revealed clear separation between SD and NSD samples, indicating a consistent hypothalamic transcriptional signature associated with sleep deprivation.

Across the hypothalamus, 23 genes were differentially expressed following sleep deprivation (Table 1). Genes with the strongest statistical support (lowest FDR values) included components of intracellular signaling and immune regulatory pathways, such as Rps6ka5, Mapk1, Jun, and Map2k1 (all FDR = 0.00158). Additional differentially expressed genes included Cd4, Plcb1, Map2k4, Nr3c1, Mef2d, Gnas, Hdac4, and Cdc42 (FDR range: 0.022–0.026). Immune signaling-related genes, such as C1qb (FDR = 0.029), Prkcb1 (FDR = 0.030), Pdgfa (FDR = 0.0399), Il1rap (FDR = 0.0424), and Tollip (FDR = 0.0488) were also differentially expressed. Full differential expression statistics, including log₂ fold changes, *p*-values, and adjusted FDR values, are provided in Table 1.

Volcano plot visualization confirmed the magnitude and statistical significance of sleep deprivation–induced transcriptional changes in the hypothalamus (Figure 3B). Of the 185 genes analyzed, 23 genes (12.8%) met the significance threshold (FDR < 0.10) and clustered above the threshold line. These genes exhibited both positive and negative log₂ fold changes, indicating bidirectional regulation of inflammatory and signaling pathways.

These differential gene expression patterns were region specific. We quantified the number of genes significantly altered by sleep deprivation across all sampled brain regions (Figure 3C). The majority of transcriptional changes were restricted to the hypothalamus, with 23 genes meeting the significance threshold in this region, whereas only one gene (Fos) was differentially expressed in the thalamus and no genes met the significance threshold in the hippocampus, motor cortex, or S1BF (Figure 3C; Table 1). These findings indicate that sleep deprivation induces a highly region-specific inflammatory transcriptional response. Subsequent analyses were therefore restricted to hypothalamic differentially expressed genes.

To determine whether the transcriptional response observed in the hypothalamus extended to other brain regions, we compared the effect sizes of hypothalamus-significant genes across all sampled regions. While these genes exhibited large expression changes in the hypothalamus, effect sizes in the thalamus, hippocampus, motor cortex, and S1BF clustered near zero, indicating that sleep deprivation–induced transcriptional changes were largely restricted to the hypothalamus (Supplementary Figure S1).

### Sleep deprivation alters coordinated inflammatory pathways and signaling pathways in the hypothalamus

To determine whether the transcriptional differences observed in the hypothalamus reflected coordinated biological processes, we performed gene set enrichment analysis (GSEA) using Kyoto Encyclopedia of Genes and Genomes (KEGG) pathway annotations applied to ranked differential expression results comparing sleep deprivation (SD) and no sleep deprivation (NSD). This approach incorporates all genes in the NanoString panel, enabling identification of pathway-level changes independent of differential expression thresholds.

**Figure 4:**
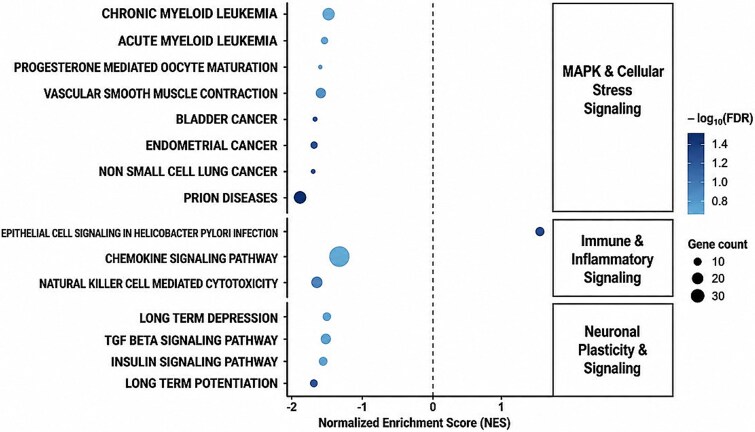
KEGG pathway enrichment analysis of hypothalamic transcriptional responses to sleep deprivation. Gene set enrichment analysis (GSEA) was performed using ranked differential expression results from the hypothalamus comparing sleep deprivation (SD) and no sleep deprivation (NSD) conditions, incorporating all genes in the NanoString panel with Kyoto Encyclopedia of Genes and Genomes (KEGG) pathway annotations and fast gene set enrichment analysis (fgsea). Enriched pathways were grouped into major functional categories, including MAPK and cellular stress signaling, immune and inflammatory signaling, and neuronal plasticity and signaling. Each point represents a KEGG pathway. Point size indicates the number of genes within each pathway represented in the NanoString panel (not restricted to differentially expressed genes), and color represents the significance of enrichment (−log₁₀ false discovery rate [FDR]). The x-axis shows the normalized enrichment score (NES), where negative values indicate pathways enriched among genes downregulated following sleep deprivation. Although the NanoString panel is enriched for inflammation-related genes, this analysis identifies specific signaling pathways and coordinated transcriptional programs engaged by sleep deprivation. These results reveal coordinated suppression of pathways related to cellular stress responses, immune signaling, and neuronal plasticity in the hypothalamus following sleep deprivation.

Multiple pathways were significantly enriched in response to sleep deprivation (Figure 4). Enriched pathways grouped into three major functional categories: MAPK and cellular stress signaling, immune and inflammatory signaling, and neuronal signaling pathways. These results indicate that sleep deprivation engages coordinated transcriptional programs across multiple signaling networks within the hypothalamus. Normalized enrichment scores (NES) indicated that many pathways were enriched among genes with negative fold changes, consistent with coordinated downregulation of multiple signaling pathways following sleep deprivation (Figure 4).

To identify genes contributing to pathway-level enrichment, we examined the leading-edge subsets for each pathway. Leading-edge genes represent the subset of genes within each pathway that most strongly contribute to the enrichment signal and are derived from the full ranked gene list rather than being restricted to the subset of differentially expressed genes. These genes included regulators of intracellular signaling and immune function such as Mapk1, Mapk3, Map2k1, Map2k4, Jun, Fos, Nfkb1, Traf2, Ripk1, Myd88, Il1a, Il1b, Il6, Tnf, Ccl3, and Ccl7. Leading-edge genes were grouped into broader functional categories to highlight shared biological themes across enriched pathways (Supplementary Table S1).

Together, these results demonstrate that sleep deprivation induces coordinated, pathway-level regulation of inflammatory and signaling networks that is region specific to the hypothalamus. These effects were observed in the presence of TBI, with no significant main effects of injury status.

## Discussion

This study demonstrates that acute sleep deprivation is the dominant driver of early neuroimmune transcriptional remodeling following TBI and that these effects are highly region-specific, with the hypothalamus emerging as a primary site of gene expression. Across all regions examined, sleep deprivation induced robust transcriptional changes, whereas TBI alone produced comparatively modest effects at 6 hours post-injury and post-sleep deprivation, and no interaction between TBI and sleep deprivation was detected. These findings provide new insight into the temporal dynamics of neuroimmune activation after injury and identify sleep deprivation as an early and active modulator of the post-injury molecular environment.

The limited transcriptional response observed following TBI at 6 hours post-injury is consistent with the well-established biphasic nature of TBI-induced neuroinflammation. While the primary injury triggers immediate cellular disruption and release of damage-associated molecular patterns, the full neuroinflammatory cascade, characterized by microglial activation, cytokine production, and blood–brain barrier dysfunction, typically evolves over hours to days [8, 9, 25, 26]. As such, the relatively modest transcriptional changes observed here could miss the peak inflammatory activation either before or after the 6 hour time point. In contrast, sleep deprivation engages rapid, state-dependent inflammatory signaling pathways that scale with wake duration, including cytokine accumulation (e.g. IL-1β, TNF-α) and activation of intracellular stress pathways such as NF-κB and MAPK signaling [21, 36, 42]. These distinct temporal profiles support a model in which sleep loss functions as an immediate modulator of neuroimmune signaling capable of shaping early transcriptional responses before the delayed inflammatory cascade induced by TBI fully emerges. While prior studies have frequently reported increased inflammatory signaling following sleep deprivation, the present findings suggest that the acute response to sleep loss immediately following injury may involve coordinated suppression or reorganization of multiple signaling networks rather than simple inflammatory activation.

Notably, the absence of a significant interaction between TBI and sleep deprivation suggests that, at this acute time point, sleep deprivation does not amplify TBI-induced transcriptional responses but instead dominates the molecular landscape. This finding should not be interpreted as evidence of independence between these processes, but rather as a reflection of temporal misalignment in their underlying biology. It is likely that interactions between TBI and sleep disruption become more pronounced at later time points, when injury-induced inflammation reaches peak activation and overlaps with sustained or repeated sleep disturbances. Indeed, prior work has demonstrated that post-injury sleep disruption exacerbates neuroinflammation and microglial activation at later stages [43–46], supporting a time-dependent interaction between these processes. And clinically, sleep deprivation does not worsen the outcome of experimental brain injury, rather extending some cellular markers of pathophysiology [24].

A central finding of this study is the striking regional specificity of the transcriptional response to sleep deprivation. The hypothalamus exhibited robust and coordinated changes in inflammatory gene expression, whereas other regions, including the thalamus, hippocampus, and cortex, showed minimal or no significant transcriptional alterations. Notably, many of the pathways and genes identified were downregulated following sleep deprivation, indicating that the response is not simply characterized by increased inflammatory signaling but rather reflects coordinated suppression and reorganization of inflammatory and signaling networks. This finding suggests that acute sleep deprivation does not necessarily promote inflammatory activation at this time point, but instead induces a more complex transcriptional response involving coordinated regulation of immune, stress-response, and neuronal signaling pathways. This regional pattern is consistent with the unique role of the hypothalamus as an integrative hub for sleep–wake regulation, circadian timing, and neuroimmune signaling [47]. Neuronal populations within the hypothalamus, including the ventrolateral preoptic nucleus (VLPO), hypocretin neurons of the lateral hypothalamus, and histaminergic neurons of the tuberomammillary nucleus, are continuously engaged in regulating behavioral state and are highly sensitive to both inflammatory signals and sleep pressure [31, 34, 47–49]. Prolonged wakefulness may therefore impose a disproportionate physiological demand on hypothalamic circuits, triggering adaptive transcriptional responses that include downregulation of inflammatory and signaling pathways to maintain homeostasis and prevent excessive or maladaptive neuroimmune activation.

At the molecular level, sleep deprivation altered the expression of genes involved in key intracellular signaling pathways, including MAPK cascade components (*Mapk1, Map2k1*), immediate early genes (*Jun*), and regulators of inflammatory signaling (*Il1rap, Tollip*). These genes are central nodes in pathways that coordinate cellular stress responses, immune activation, and synaptic signaling. Gene set enrichment analysis further revealed coordinated suppression of MAPK/ERK signaling, NF-κB-mediated inflammatory pathways, cytokine and chemokine signaling, complement activation, and cytoskeletal remodeling pathways. This broad suppression of canonical inflammatory and stress signaling pathways was somewhat unexpected given prior reports of increased MAPK and NF-κB activation following sleep deprivation [50, 51]. However, the present findings likely reflect a systems-level transcriptional response that integrates signals across multiple cell types within the hypothalamus. One interpretation is that acute sleep loss induces an early compensatory or adaptive phase characterized by suppression of inflammatory and stress-response signaling pathways. Such a response may function to maintain homeostasis and limit excessive neuroimmune activation during prolonged wakefulness. Alternatively, this early suppression may precede a later phase of inflammatory amplification, which would not be captured within the present 6-hour observation window. Importantly, because these analyses were performed on bulk tissue punches, the observed transcriptional patterns likely reflect the integrated response of multiple cellular populations. Cell type-specific activation of inflammatory pathways may therefore occur despite an overall reduction in pathway expression at the tissue level. Future studies employing single-cell or spatial transcriptomic approaches will be necessary to resolve these possibilities.

Importantly, the regional specificity observed here aligns with emerging evidence that sleep loss does not uniformly affect the brain, but instead produces spatially heterogeneous molecular and functional alterations. In prior work from our group, acute sleep deprivation in male mice was sufficient to induce accumulation of neurodegenerative disease-related protein variants (e.g. Aβ, tau, α-synuclein, and TDP-43) in a region-specific manner, with the greatest pathology observed in cortical and white matter regions [52]. These findings demonstrate that sleep loss alone is capable of driving pathological processes typically associated with neurodegenerative disease and further support the concept that specific brain regions exhibit differential vulnerability to sleep loss. Together with the current data, these results suggest that sleep deprivation may engage regionally distinct but mechanistically linked processes, including both transcriptional reprogramming and protein pathology, which may converge to influence long-term neurological outcomes.

The convergence of region-specific transcriptional and protein-level responses highlights sleep loss as a critical regulator of brain responses following injury. The hypothalamus, by virtue of its central role in coordinating behavioral state, circadian timing, and neuroimmune signaling, may act as an early sensor and integrator of sleep-dependent stress signals. Disruption of hypothalamic signaling could therefore propagate downstream effects across distributed neural networks, influencing both inflammatory signaling and circuit-level function. Given that hypothalamic dysfunction has been implicated in post-traumatic sleep disturbances and circadian dysregulation [15, 53–57], these findings provide a mechanistic link between acute sleep loss and neuroimmune signaling that defines post-injury pathophysiology into the chronic condition.

Several limitations should be considered when interpreting these findings. First, the analysis was restricted to a single acute time point, which limits conclusions regarding the temporal evolution of neuroinflammatory responses and potential interactions between TBI and sleep deprivation at later stages. Second, transcriptional profiling was performed at the bulk tissue level, precluding cell type-specific resolution of gene expression changes. Future studies using single-cell or spatial transcriptomic approaches will be critical for identifying the cellular populations driving these effects. Third, this study was conducted in male mice, and given known sex differences in both sleep regulation and neuroinflammatory responses [4, 45, 58], future work should incorporate female cohorts to determine whether these findings generalize across sexes. Although the study utilized a modest number of animals per group, each animal contributed multiple anatomically defined brain regions, enabling a regionally resolved analysis across the neuroaxis. The emergence of a highly localized hypothalamic transcriptional signature despite this design suggests a robust regional effect; however, future studies with larger cohorts will be important for validating these findings and detecting more subtle transcriptional changes in other brain regions.

In summary, these data identify acute sleep deprivation as a potent and region-specific regulator of early neuroimmune transcriptional responses following TBI, with the hypothalamus representing a key site of vulnerability. Rather than promoting widespread inflammatory activation, acute sleep deprivation induced coordinated alterations in inflammatory, cellular signaling, and neuronal pathways, many of which were characterized by reduced expression. These findings suggest that sleep loss may engage adaptive or compensatory transcriptional programs during the acute post-injury period while simultaneously reshaping the molecular environment in ways that could influence subsequent injury responses. Future studies examining additional time points and cell type-specific transcriptional responses will be necessary to determine whether these early changes remain protective, become maladaptive, or contribute to later neuroinflammatory processes. Collectively, these findings establish sleep as a major regulator of early neuroimmune signaling after injury and support further investigation into how sleep-dependent mechanisms influence recovery following TBI.

## Supplementary Material

Supplementary_Material_zpag076

## Data Availability

The data supporting the conclusions of this manuscript will be made available upon reasonable request.
