# Supplementary material for: Acute sleep deprivation alters hypothalamic neuroinflammatory signaling independent of traumatic brain injury in the mouse
Source: Sleep Adv. 2026 Jul 13;7(3):zpag076. doi: 10.1093/sleepadvances/zpag076 (PMC13480474; doi:10.1093/sleepadvances/zpag076)
Supplement: Supplementary file 1 [file supplementary_material_zpag076.docx]

**Acute sleep deprivation alters hypothalamic neuroinflammatory signaling independent of traumatic brain injury in the mouse**

**Francois Labadie^1^, Grant S. Mannino^1,2^, Jonathan Lifshitz^3,4^, and Rachel K. Rowe^1*^**

^1^ Department of Integrative Physiology, University of Colorado Boulder, Boulder, CO, USA

^2^ Stanford University School of Medicine, Neurosciences Interdepartmental Program, Stanford, CA, USA

^3^ Physical Medicine and Rehabilitation, University of Michigan, Ann Arbor, MI, USA

^4^ University of Michigan Concussion Center, Ann Arbor, MI, USA

*** Correspondence:**
Rachel K. Rowe, Ph.D.

Assistant Professor

Department of Integrative Physiology

University of Colorado Boulder

2860 Wilderness Place

Boulder, CO 80301

**SUPPLEMENTARY FIGURES LEGENDS AND TABLES**

**Supplementary Figure 1. Regional distribution of sleep deprivation effect sizes for hypothalamus-significant genes.** Each point represents the log₂ fold change (sleep deprivation [SD] vs no sleep deprivation [NSD]) for genes that were differentially expressed in the hypothalamus (FDR < 0.10). Effect sizes for the same genes are shown across hypothalamus, thalamus, hippocampus, motor cortex, and primary somatosensory cortex (S1BF). While the hypothalamus exhibits large transcriptional shifts, effect sizes in other regions cluster near zero, indicating that sleep deprivation-induced inflammatory gene expression changes are highly region specific.

**Supplementary Figure 1 Alt Text:** Scatter plot showing that sleep deprivation-induced gene expression effect sizes were largest in the hypothalamus and clustered near zero in other brain regions, indicating strong regional specificity.

| **Pathway Category** | **Direction** | **Leading Edge Genes** |
| --- | --- | --- |
| MAPK / ERK Signaling | ↓ | Mapk1, Mapk3, Map2k1, Map2k4, Map2k6, Raf1, Rps6ka5, Jun, Fos, Mapk14 |
| NF-κB & Innate Immune Signaling | ↓ | Nfkb1, Traf2, Ripk1, Ripk2, Myd88, Tollip |
| Complement Cascade | ↓ | C1qa, C1qb, C3, C3ar1, C9, Cfb, Hc, Masp2 |
| Cytokine & Chemokine Signaling | ↓ | Il1a, Il1b, Il1rap, Il1r1, Il6, Il18, Tnf, Ccl3, Ccl7, Ccl11 |
| TGF-β Signaling | ↓ | Tgfb1, Tgfb2, Tgfbr1 |
| Cell Adhesion & Cytoskeletal Remodeling | ↓ | Cdc42, Rac1, Rhoa, Itgb2, Cfl1, Ppp1r12b |
| Stress & Glucocorticoid Signaling | ↓ | Gnas |
| Synaptic & Plasticity Signaling | ↓ | Grb2, Plcb1, Ptk2, Rapgef2, Mef2c |

**Supplementary Table 1:** Genes listed represent the leading-edge subset identified by fast gene set enrichment analysis (fgsea) of KEGG pathways using ranked differential expression results from hypothalamic tissue comparing sleep deprivation (SD) and no sleep deprivation (NSD). The leading-edge subset includes genes that contribute most strongly to the enrichment signal for each pathway. Pathways were grouped into broader functional categories to illustrate shared biological themes. Direction arrows (↓) indicate pathways enriched among genes downregulated following sleep deprivation. These genes collectively highlight coordinated changes in cellular stress responses, immune and inflammatory signaling, cytoskeletal remodeling, and neuronal signaling pathways in the hypothalamus following sleep deprivation. Abbreviations: MAPK/ERK signaling (mitogen-activated protein kinase / extracellular signal-regulated kinase signaling), NF-κB signaling (nuclear factor kappa-light-chain-enhancer of activated B cells signaling), TGF-β signaling (transforming growth factor beta signaling), and cytokine and chemokine signaling pathways (immune signaling pathways mediated by cytokines and chemokines).
